# Cloning and characterization of a novel oocyte-specific gene encoding an F-Box protein in rainbow trout (Oncorhynchus mykiss)

**DOI:** 10.1186/1477-7827-11-86

**Published:** 2013-09-04

**Authors:** Lei Wang, Swamy K Tripurani, Warapond Wanna, Caird E Rexroad, Jianbo Yao

**Affiliations:** 1 Division of Animal and Nutritional Sciences, West Virginia University, Morgantown, WV 26506, USA; 2 National Center for Cool and Cold Water Aquaculture, Kearneysville, WV 25430, USA; 3 Current address: Center for Genomics and Bioinformatics Research, Faculty of Science, Prince of Songkla University, Hat-Yai Songkhla 90112, Thailand

**Keywords:** F-Box protein, Oocyte, Vitellogenesis, Rainbow trout

## Abstract

**Background:**

Oocyte-specific genes play critical roles in oogenesis, folliculogenesis and early embryonic development. The objectives of this study were to characterize the expression of a novel oocyte-specific gene encoding an F-box protein during ovarian development in rainbow trout, and identify its potential interacting partners in rainbow trout oocytes.

**Methods:**

Through analysis of expressed sequence tags (ESTs) from a rainbow trout oocyte cDNA library, a novel transcript represented by ESTs only from the oocyte library was identified. The complete cDNA sequence for the novel gene (named *fbxoo*) was obtained by assembling sequences from an EST clone and a 5′RACE product. The expression and localization of *fbxoo* mRNA and protein in ovaries of different developmental stages were analyzed by quantitative real time PCR, immunoblotting, *in situ* hybridization and immunohistochemistry. Identification of Fbxoo binding proteins was performed by yeast two-hybrid screening.

**Results:**

*fbxoo* mRNA is specifically expressed in mature oocytes as revealed by tissue distribution analysis. The *fbxoo* cDNA sequence is 1,996 bp in length containing an open reading frame, which encodes a predicted protein of 514 amino acids. The novel protein sequence does not match any known protein sequences in the NCBI database. However, a search of the Pfam protein database revealed that the protein contains an F-box motif at the N-terminus, indicating that Fbxoo is a new member of the F-box protein family. The expression of *fbxoo* mRNA and protein is high in ovaries at early pre-vitellogenesis stage, and both *fbxoo* mRNA and protein are predominantly expressed in early pre-vitellogenic oocytes. Several proteins including tissue inhibitor of metalloproteinase 2 (Timp2) were identified as potential Fbxoo protein binding partners.

**Conclusions:**

Results suggest that the novel oocyte-specific F-box protein may play an important role in early oocyte development by regulating other critical proteins involved in oogenesis in rainbow trout.

## Background

The finely orchestrated development and maturation of the oocyte have been the focus of many studies in which essential oocyte-specific genes have been identified [1,2]. Examples of such genes include factor in germline alpha (*Figla*), growth differentiation factor 9 (*Gdf9*) and newborn ovary homeobox (*Nobox*). Deficiency of these essential genes in mice leads to the failure of primordial follicle formation or the disruption of early follicular development [3-5].

In fish, the initial stages of oogenesis are featured by a short period of intense RNA synthesis prior to vitellogenesis, which is a principal event responsible for the enormous growth of oocytes [6]. Studies of oocyte-specific genes associated with oogenesis and vitellogenesis in fish are limited. In zebrafish, an oocyte-specific gene, *zorg* (zebrafish oogenesis related gene), has been reported and its expression profiles in different stages of oocytes suggests a role of this gene in the formation of female germ cells [7]. Another zebrafish gene, *zvep* (zebrafish vitelline envelope protein), was shown to be specifically expressed in zebrafish ovary and brain [6]. Analysis of zvep expression in ovaries shows that, as a component of the vitelline envelope, zvep is synthesized during early development of oocytes. Based on analysis of EST sequences from a rainbow trout oocyte cDNA library, we have previously identified two novel oocyte-specific genes (*oorp-t* and *rtgst-1*) in rainbow trout. *oorp-t* encodes a protein with a conserved oxysterol binding protein (OSBP) domain, suggesting its role in the synthesis, transportation and metabolism of lipids during oogenesis [8]. *rtgst-1* is a noncoding mRNA-like transcript and its predominant expression in early previtellogenic oocytes suggests a crucial role of this transcript in the differentiation and/or early development of oocytes [9].

F-box proteins are defined as proteins containing at least one F-box domain (a motif of approximately 50 amino acids). They are one of the components of the SCF complex (Skp1, Cullin1 and F-box), the most important ubiquitin E3 ligase, which is known to have various functions such as control of cell cycle, signal transduction, transcription and post-translational modification through ubiquitin modification system across several species [10-12]. The first SCF complex pathway was identified in yeast and it appears to recognize and ubiquitinate only phosphorylated substrates. Apart from regulating protein phosphorylation networks and proteolytic degradation, SCF complex also regulates a great number of cellular pathways by shifting substrate specific adaptor subunits, the F-box proteins [13]. F-box proteins recognize the specific target protein substrates through the secondary motifs on the carboxyl-terminal of F-box proteins [12]. The most common secondary motifs that F-box proteins possess include WD repeats and leucine-rich repeats. Based on different secondary motifs they have, F-box proteins are classified as FBXW (contain WD repeats), FBXL (contain leucine-rich repeats) and FBXO (F-box only) [14].

In this study, we report the identification of a novel oocyte-specific F-box protein (named Fbxoo) and the characterization of its mRNA and protein expression during oogenesis in rainbow trout. We show that Fbxoo is predominantly expressed in early pre-vitellogenic oocytes and interacts with several proteins including tissue inhibitor of metalloproteinase 2 (Timp2), a known important factor for follicular development. Results suggest that Fbxoo may play a distinct and important role in the early development of oocytes in rainbow trout.

## Methods

### Fish sample collection

Ovarian samples were collected from female rainbow trout of different stages of development. The stages of ovarian development in fish were determined based on morphological characteristics and the size of the oocytes as described previously [15]. The five stages of ovarian development include: early pre-vitellogenesis (≤ 0.5 mm), late pre-vitellogenesis (≤ 0.65 mm), early-vitellogenesis (0.65-1.1 mm), med-vitellogenesis (1.1-2.1 mm) and late-vitellogenesis (2.1-4.0 mm). Mature oocytes were collected from spawning fish. Fertilized oocytes were incubated at 13°C in a flow through system using a photoperiod of 12 h light-12 h dark. Embryonic samples were collected at day 0, 4 and 7 after fertilization. Various tissue samples including heart, gill, testis, intestines, muscle, brain, kidney, liver, spleen and skin were collected from mature fish. All samples were quick frozen in liquid nitrogen and stored in −80°C until use.

### RNA isolation and cDNA synthesis

Extraction of total RNA from all samples was carried out using Tri-Reagent (Ambion, Austin, TX) according to the manufacturer’s protocol. Isolated total RNA was treated with DNase I (Promega Madison, WI) before cDNA synthesis. About three μg of DNase-treated total RNA were used for first strand cDNA synthesis (20-μl reaction) using Oligo (dT)_18_ primer and SuperScript III reverse transcriptase (Invitrogen, Carlsbad, CA). Negative control reverse transcription reactions (without reverse transcriptase) were conducted to confirm no genomic DNA contamination in the RNA preparations.

### Reverse transcription polymerase chain reaction (RT-PCR)

RT-PCR was used to analyze the tissue distribution of *fbxoo* mRNA. The cDNA samples were used as PCR templates to amplify a 524 bp fragment of *fbxoo* mRNA using gene specific primers (Table 1). PCR was performed in a 25-μl reaction mix with the condition of a 5 min denaturation at 94°C followed by 30 cycles of 94°C for 30 sec, 58°C for 45 sec, and 72°C for 30 sec, and a final extension at 72°C for 10 min. Trout *β-actin* gene (*actb*) was used as an internal control for RNA quantity.

**Table 1:** Primers used in this study

**Primer name**	**Primer sequence 5′-3′**	**Application**
Fbxoo-F851	GACATCAGATACCTGGCCTCA	RT-PCR
Fbxoo-R1374	TGGGAGATCTTGGTGTCAAAC	RT-PCR
Fbxoo-SP1-50	GCGAGAACTGTCCCTGCAAC	5′RACE
Fbxoo-SP2-105	CGTCCATGTGTGTCCTCACTT	5′RACE
Fbxoo-SP3-136	CGTCACTTAAGTCTAGTATCCC	5′RACE
Fbxoo-F1436	CTCTACCTGGAGGCAGTGAAA	Real time PCR
Fbxoo-R1567	GAGTGCTCAATCTGCGTTAGC	Real time PCR
HistoneH2A-F	TCCCCAAGAAGACTGAGAAGG	Real time PCR (control)
HistoneH2A-R	TTTGTTGAGCTAGGTGGTTGG	Real time PCR (control)
Fbxoo-Y2H-F	ATGTCGACTTATGGCACTTCGT	Yeast two hybridization
Fbxoo-Y2H-R	ATATGTCGACCTAGCTGAC	Yeast two hybridization

### 5′Rapid amplification of cDNA end (5′RACE)

To obtain the 5′end of the cDNA sequence, 5′RACE was performed using the second generation 5′/3′ RACE kit (Roche Diagnostics, Indianapolis, IN) following the manufacturer’s protocol with a minor modification. The first strand cDNA was synthesized using random hexamers instead of the kit provided d(T) primer. Nested PCR reactions were performed using gene specific primers (Table 1) in conjunction with the d(T) anchor primers provided by the kit. The final RACE product was cloned into pGEM T-easy vector (Promega) and sequenced.

### Northern blot analysis

Poly(A)+RNA isolated from oocyte total RNA was separated by electrophoresis on a 1% denaturing agarose gel containing 2.2M formaldehyde (Promega), and transferred to a Hybond N+ nylon membrane (Amersham Biosciences, Piscataway, NJ). A DIG-labeled DNA probe was synthesized by PCR using a plasmid containing a 524 bp fragment of the *fbxoo* cDNA and the PCR DIG probe synthesis kit (Roche Diagnostics). Pre-hybridization of the membrane was performed using DIG Easy Hyb solution (Roche Diagnostics). Ten μl of the probe was added to DIG Easy Hyb solution and the hybridization was carried out overnight at 68°C. The membrane was washed under stringent conditions followed by incubation in 5% blocking solution (Roche Diagnostics) for 30 min. The membrane was then incubated in 1:10000 diluted alkaline phosphatase conjugated anti-DIG antibody (Roche Diagnostics) for another 30 min. After washing, the hybridized probe was detected with the chemiluminescent substrate CSPD (Roche Diagnostics).

### Generation of anti-Fbxoo antibody

A polyclonal antibody against Fbxoo was generated by immunizing rabbits with a 15 amino acid synthetic peptide (CQKHSKRKVVSWGGT) of Fbxoo protein. The antibody was prepared and affinity purified commercially by GenScript Corporation (Piscataway, NJ).

### Western blot analysis

Frozen ovarian samples were homogenized in T-PER protein extraction buffer containing Halt™ Protease inhibitor cocktail (Thermo Fisher Scientific, Waltham, MA). Protein concentrations of the samples were determined using the Coomassie protein assay kit (Thermo Fisher Scientific). Five μg of protein from each sample were loaded on a Tris–HCl ready gel (Bio-Rad, Hercules, CA) and the electrophoresis was run in 1X Tris/Glycine/SDS running buffer for 2 h. Proteins were then transferred onto a PVDF membrane in 1 X transfer buffer (Tris/Glycine/SDS/methanol). After blocking overnight at 4°C in blocking solution (5% non-fat dry milk in Tris-buffered saline containing 0.1% Tween-20, TBST), the membrane was incubated with 1:100 diluted affinity purified rabbit anti-Fbxoo polyclonal antibody for 2 h. After washing for 3 times with TBST, the membrane was incubated with goat-anti-rabbit IgG antibody conjugated to horseradish peroxidase enzyme for 2 h. Detection of the proteins was performed using the SuperSignal West Pico Chemiluminescent substrate (Thermo Fisher Scientific).

### In situ hybridization

Paraformaldehyde-fixed ovarian samples were embedded with melted paraffin, sectioned (5 μm), and mounted onto glass slides. The sections were de-paraffinized followed by washing with PBS for 3 times. The sections were then digested with proteinase K (2 μg/ml) for 15 min at 37°C and acetylated in 0.25% (v/v) acetic anhydride (prepared in 0.1 M triethanolamine, pH 8.0). The slides were washed with 4X SSC for 2 times followed by incubatation in 50% (v/v) deionized formamide (prepared in 2X SSC) at 42°C for 30 min. About 100 μl of ready-to-use prehybridization solution (Biochain, Newark, CA) was applied to each slide and incubated at 50°C for 4 h in a plastic box with water soaked paper towels to avoid evaporation. Hybridization of the slides was performed using RNA probes (both sense and antisense) prepared with a DIG RNA labeling kit (Roche Diagnostics) at 45°C overnight. After hybridization, the slides were treated with RNase A (20 μg/ml) for 30 min at 37°C and washed 3 times with 0.1X SSC at 42°C. Hybridized probes were detected with an alkaline phosphatase conjugated anti-DIG antibody (Roche Diagnostics) and the corresponding substrate, NBT/BCIP (Roche Diagnostics).

### Immunohistochemistry

The paraffin slides were de-paraffinized and rehydrated followed by treatment with 0.3% H_2_O_2_ in methanol to quench the endogenous peroxidase activity. The ABC Peroxidase Staining kit and Metal Enhanced DAB Substrate kit (Thermo Fisher Scientific) were used to detect and visualize the protein signals according to the manufacturer’s instructions. The rabbit anti-Fbxoo polyclonal antibody was used as a primary antibody in the analysis.

### Real time polymerase chain reaction

The expression of *fbxoo* mRNA during vitellogenesis and early embryonic development was quantified using quantitative real time PCR. PCR primers for *fbxoo* gene and the endogenous control gene (*histone H2A*) are shown in Table 1. Quantitative real time PCR was performed for each cDNA sample in duplicate on a Bio-Rad iCycler iQ Real-Time PCR Detection System using iQ™ SYBR^®^ Green Supermix (Bio-Rad) in a 25-μl reaction volume containing cDNA generated from 0.1 μg of total RNA. Standard curves for the *fbxoo* gene and the control gene were constructed using 10-fold serial dilutions of the corresponding plasmids. Standard curves for both *fbxoo* and the control gene were run on the same plate with the samples. Threshold lines were adjusted to intersect amplification lines in the linear portion of the amplification curves and cycles to threshold (Ct) were recorded. For each sample, the quantities of the *fbxoo* mRNA and the control gene mRNA were determined from the appropriate standard curves. To obtain a normalized value of *fbxoo* gene, the quantity of *fbxoo* mRNA was divided by the quantity of *histone H2A* mRNA. One way analysis of variance (ANOVA) was performed on normalized gene expression values using a statistical analysis package, SigmaStat version 3.11 (Aspire Software International, Leesburg, VA). The expression of *fbxoo* mRNA was then shown as relative fold changes.

### Yeast two hybridization

The Matchmaker Two-Hybrid System (Clontech Laboratories, Mountain View, CA) was used to identify proteins interacting with Fbxoo according to the manufacturer’s instructions. To generate the bait expression vector, the coding region of *fbxoo* was cloned in frame in the pGBKT7 vector (Clontech Laboratories) at the NdeI and SalI sites. Yeast AH109 competent cells were co-transformed with a SMART PCR amplified oocyte cDNA library, a linearized pGADT7 plasmid and the bait expression plasmid (pGBKT7-Fbxoo). Yeast cells were plated on synthetic dropout selection medium lacking histidine, leucine and tryptophan (Med dropout plate: SD/-his/-leu/-try) and incubated at 30°C for 3 days. Single colonies (>2 mm) were selected and streaked on fresh high dropout plates (High dropout plate: SD/-ade/-his/-leu/-try). Plasmid was isolated from liquid culture of high dropout medium from single colonies and used to transform *E. coli* competent cells using ampicillin as antibiotic to select for pGADT7 resistant clones. The ampicillin resistant plasmids were sequenced and the sequences were used to BLAST the GenBank database. To confirm the screening result, the rescued plasmids (expressing both BD and AD binding proteins) were retransformed into host strain and plated on high dropout plates. The transformants were tested for β-galactosidase activity by both the filter lift assay and the yeast β-galactosidase liquid assay using CPRG (cholorophenol red-β-d-galacto-pyranoside) according to the manufacturer’s instructions. The β-galactosidase units were calculated using the following formula: β-galactosidase units = 1000x OD_578_/ (t x V x OD_600_). Where OD_578_ is the absorbance of cholorophenol red and OD_600_ is the cell density at the start point; t is elapsed time (in minute) of incubation; V is 0.1x concentration factor. 1 unit of β-galactosidase is defined as the amount that hydrolyzes 1 μmol of CPRG to cholorophenol and d-galactose per minute per cell.

## Results

### Cloning and sequence analysis of fbxoo cDNA

**Figure 1:**
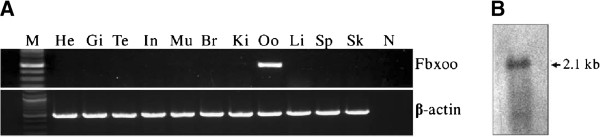
**Analysis of *fbxoo *mRNA expression. A**. Tissue distribution of *fbxoo* mRNA assessed by RT-PCR. Trout tissues tested include heart (He), gill (Gi), testis (Te), intestine (In), muscle (Mu), brain (Br), kidney (Ki), oocyte (Oo), liver (Li), spleen (Sp) and skin (Sk). M: DNA marker; N: negative control. Trout β-actin (*actb*) was used as a control for RNA quality. **B**. Northern blot analysis of *fbxoo* transcript.

Through analysis of expressed sequence tags (ESTs) from a rainbow trout oocyte cDNA library, we identified a novel transcript, which is represented by multiple ESTs derived from the oocyte cDNA library. Analysis of tissue distribution of the novel transcript by RT-PCR revealed that the novel gene is only expressed in mature oocytes but not in testis and 9 somatic tissues examined (Figure 1A). Northern blot analysis detected a single band of ~2.1 kb for this transcript in oocytes (Figure 1B). The longest EST clone was retrieved from the library and the insert of the clone was completely sequenced by primer walking. The final cDNA sequence for the novel gene was obtained by assembly of the sequence of the EST clone and a 5′end sequence from a 5′RACE product. The complete cDNA is 1,996 bp in length, which has been deposited in the GenBank with the accession number: HQ201417.

**Figure 2:**
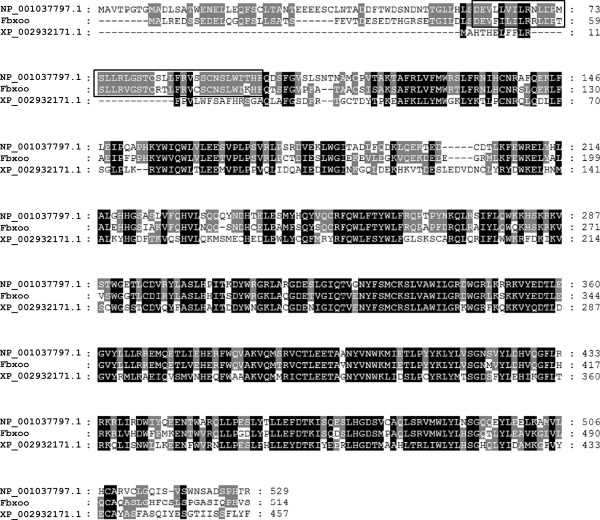
**Multiple alignment of amino acid sequences of Fbxoo protein with similar proteins from other species.** The alignment was performed with the rainbow trout Fbxoo protein, a hypothetical protein [GenBank: NP_001037797.1] from zebrafish and a predicted hypothetical protein [GenBank: XP_002932171.1] from Xenopus tropicalis using ClustalW. The F-box domain is boxed.

Analysis of the novel cDNA sequence showed that it contains a 13 bp 5′untranslated region (5′UTR), an open reading frame (ORF) of 1,545 bp and a 3′untranslated region (3′UTR) of 438 bp (Additional file 1: Figure S1). There is a typical polyadenylation signal sequence (AATAAA) and a cytoplasmic polyadenylation element (TTTTTAA) in the 3′UTR. The ORF of the cDNA encodes a protein of 514 amino acids with a predicted molecular weight of 60,012 Da. A search of the Pfam database revealed that the novel protein contains an F-box domain located at the N-terminus from residue 46 to 85. The protein does not appear to contain any of the secondary motifs such as leucine-rich repeats (LRR) and WD repeats that are commonly present in the family of F-box proteins. Therefore, we named the novel protein as Fbxoo (F-box only, oocyte specific). Further analysis revealed that Fbxoo protein contains a sumoylation consensus motif (LK_190_F). BLAST analysis showed that Fbxoo protein does not match any known proteins in the GenBank database. However, it shows sequence similarity to a zebrafish hypothetical protein [GenBank: NP_001037797.1] and a predicted hypothetical protein [GenBank: XP_002932171.1] in *Xenopus tropicalis* (Figure 2). Fbxoo protein shares higher sequence identity (~69%) with the zebrafish protein than the Xenopus protein, which appears missing the conserved F-box domain.

### Expression of Fbxoo mRNA and protein during vitellogenesis and early embryogenesis

**Figure 3:**
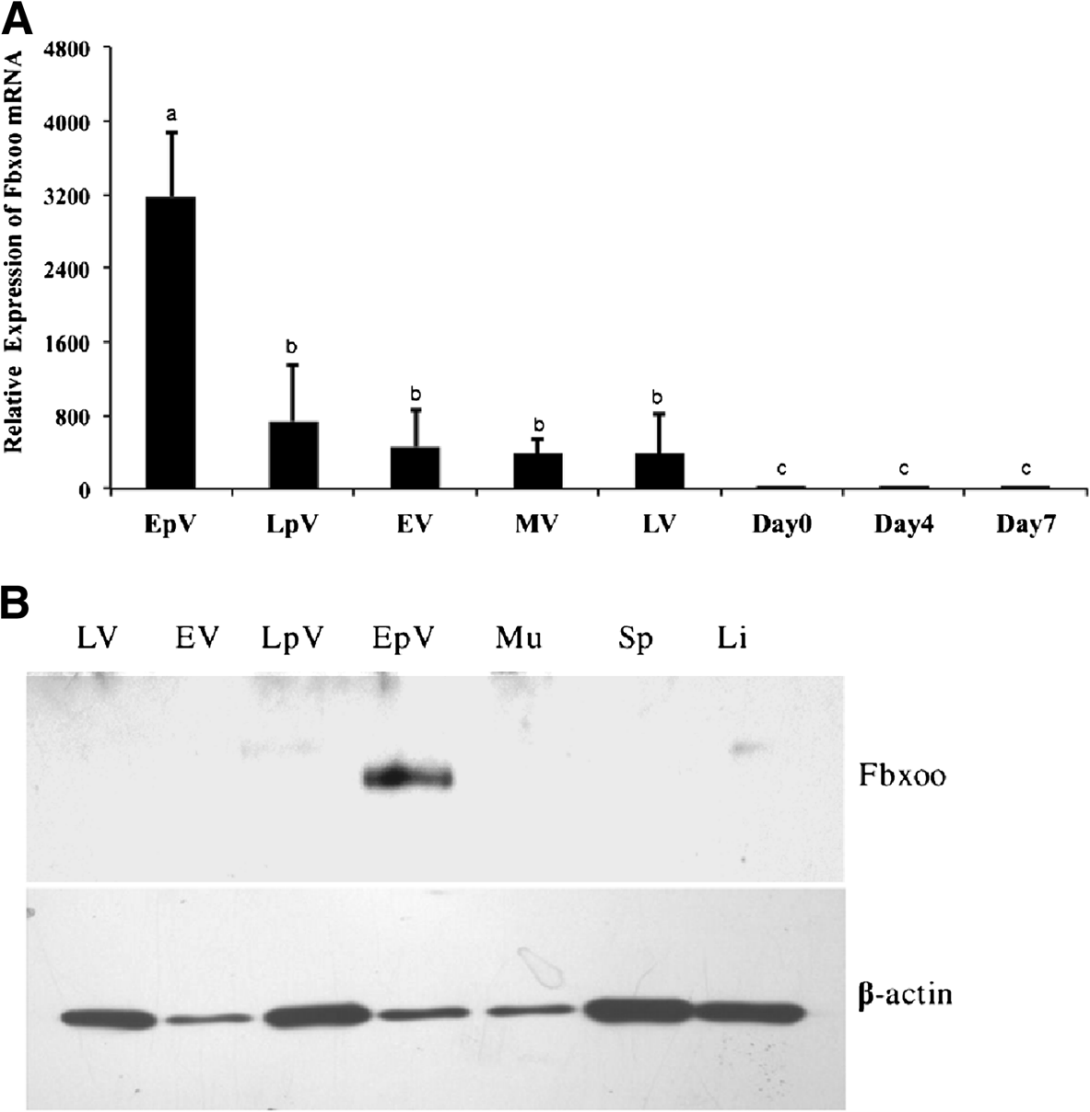
**Expression of *fbxoo *mRNA and protein during ovarian and embryonic development. A**. Quantitative analysis of *fbxoo* mRNA expression during ovarian development and early embryogenesis by real time PCR. Ovarian samples from different developmental stages including early pre-vitellogenesis (EpV), late pre-vitellogenesis (LpV), early vitellogenesis (EV), mid-vitellogenesis (MV) and late-vitellogenesis (LV) were used in the analysis. **B**. Western blot analysis of Fbxoo protein expression in ovaries of different developmental stages and selected somatic tissues including muscle (Mu), spleen (Sp) and liver (Li). Rainbow trout β-actin (Actb) serves as a control.

To determine the expression profile of *fbxoo* mRNA during vitellogenesis, ovarian samples collected from five developmental stages of vitellogenesis were subjected to quantitative real time PCR analysis. As shown in Figure 3A, *fbxoo* transcript is abundantly present in ovaries at early previtellogenesis stage but much less in ovaries at subsequent stages of development. The expression of *fbxoo* mRNA is also detected in mature unfertilized oocytes (day 0) and early embryos (day 4 and day 7), but at much lower levels relative to the developing ovaries.

Using a polyclonal antibody raised in rabbit against a synthetic peptide of Fbxoo protein, the expression of Fbxoo protein in ovaries of different developmental stages was determined by Western blot analysis. As shown in Figure 3B, the expression of Fbxoo protein can be detected in ovaries at early pre-vitellogenesis, but not in ovaries at other stages of development, nor in any of the somatic tissues tested.

### Localization of Fbxoo mRNA and protein in oocytes of different developmental stages

**Figure 4:**
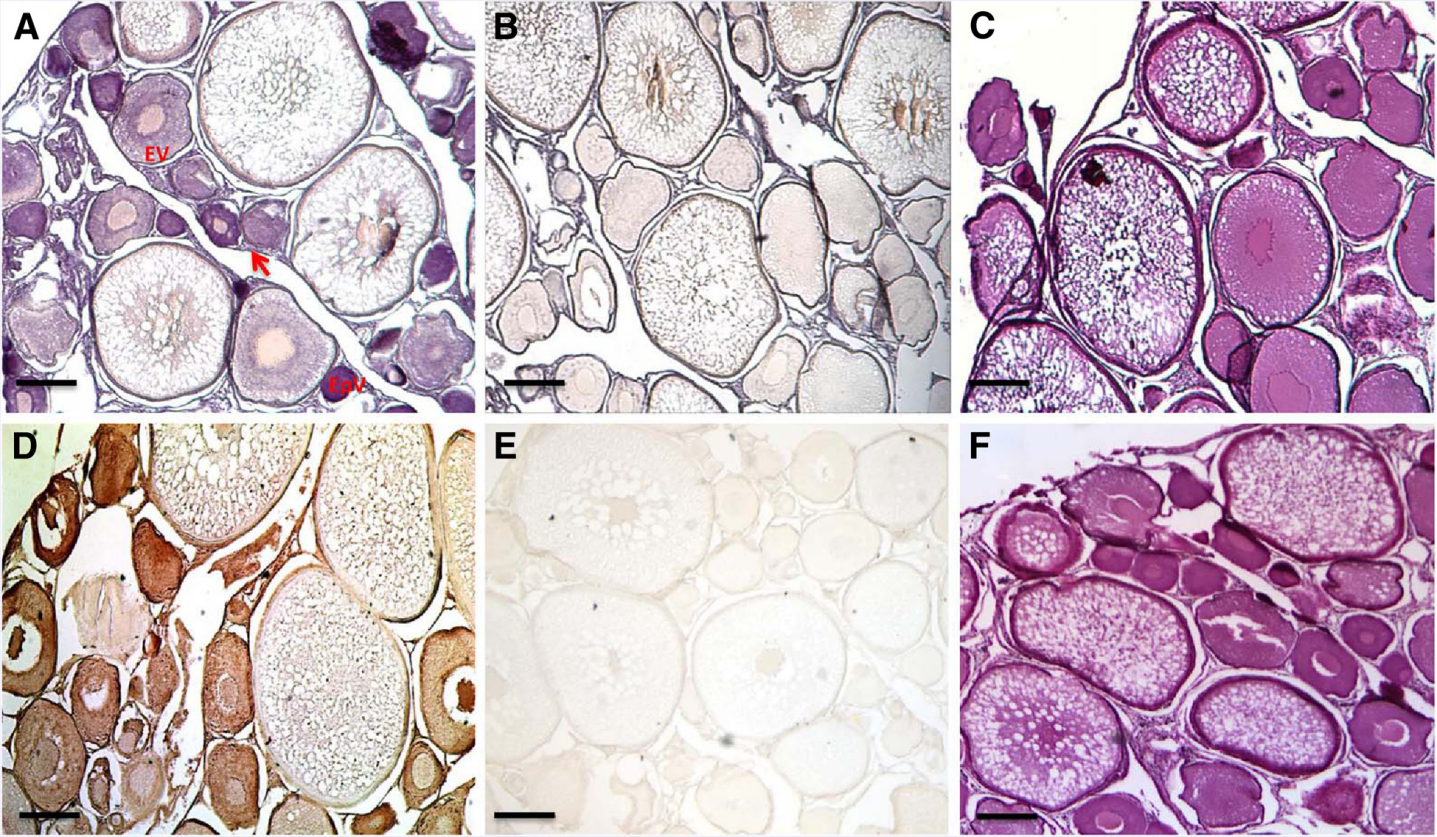
**Localization of *fbxoo *mRNA and protein in oocytes of different developmental stages. A**. *In situ* hybridization analysis using antisense RNA probe showing predominant expression of *fbxoo* mRNA in early stage oocytes. **B**. A control section hybridized with the sense probe showing no significant hybridization signal. **D**. Detection of strong Fbxoo protein expression signal in early pre-vitellogenic and early vitellogenic oocytes by immunohistochemistry using anti-Fbxoo antibody. **E**. A negative control section without primary antibody. **C** and **F**. Ovarian sections stained with Hematoxylin and Eosin to show oocytes of different vitellogenic stages. EV: early vitellogenic oocyte; EpV: early pre-vitellogenic oocyte. The arrow indicates granulosal cell layer. The scale bars indicate 300 μm.

*In situ* hybridization was performed on rainbow trout ovarian sections to determine the localization of *fbxoo* transcript. Strong hybridization signals (blue staining) were detected in the cytoplasm of small oocytes at early pre-vitellogenesis and early vitellogenesis stages (Figure 4A). There were less or no detectable signals in large oocytes of later developmental stages. No specific hybridization signal was detected in oocytes of different developmental stages in the ovarian section hybridized with the sense RNA probe (Figure 4B). Immunohistochemical analysis showed that Fbxoo protein is abundantly present in early pre-vitellogenic and early vitellogenic oocytes (Figure 4D), but not, or to much less extent, in later stage oocytes. No immunochemical staining was detected in the negative control section (Figure 4E). Ovarian sections stained with Hematoxylin and Eosin are shown in Figure 4C and 4F.

### Identification of Fbxoo binding proteins

**Figure 5:**
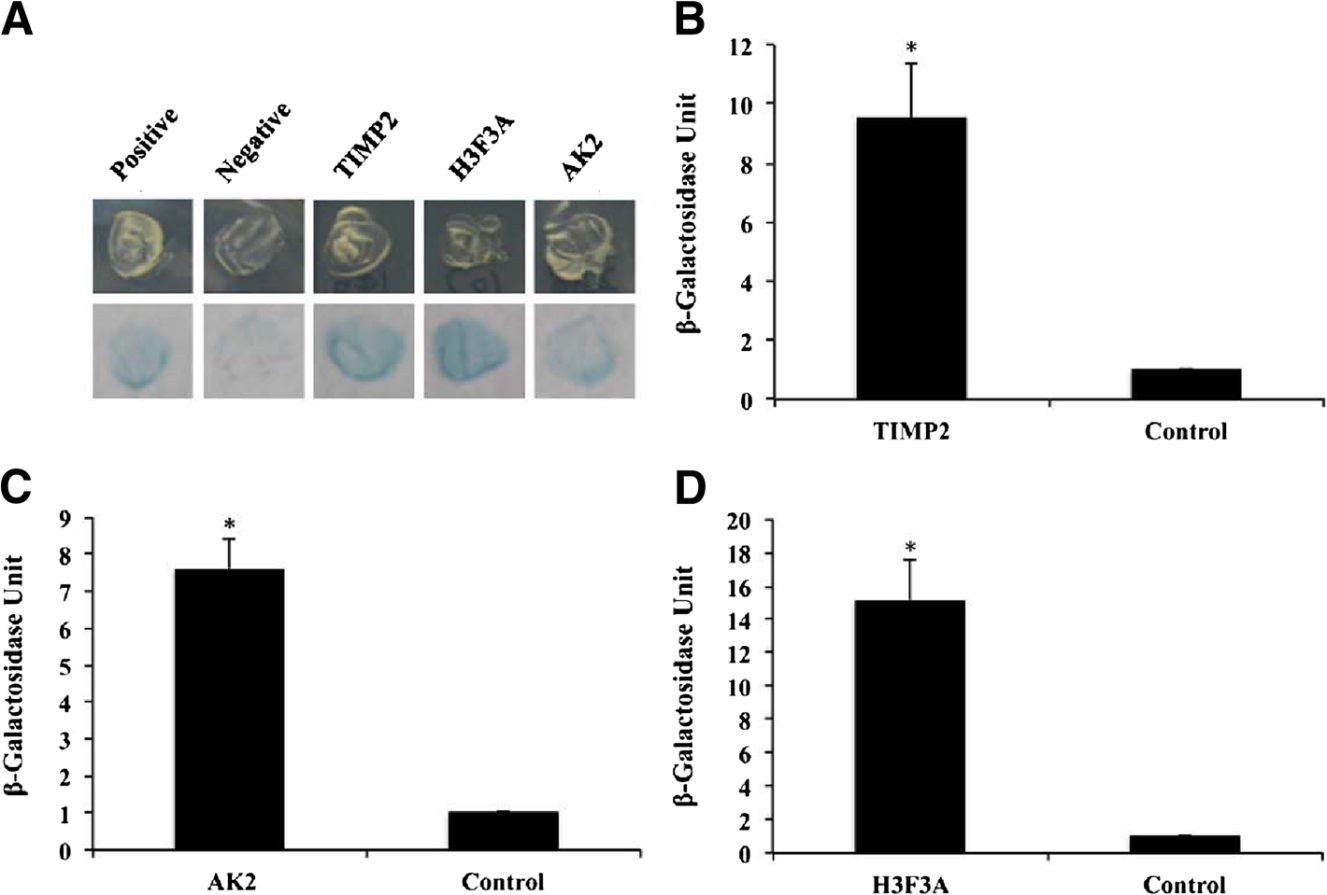
**Interaction of Fbxoo with its binding proteins identified by yeast two-hybrid screening. A**. Colony-lift filter assays showing β-galactosidase activities in yeast cultures expressing both Fbxoo protein and its interacting proteins (Timp2 or Ak2 or H3f3a). **B**-**D**. Yeast β-galactosidase liquid assays confirming interactions between Fbxoo and its binding proteins including Timp2 **(B)**, Ak2 **(C)** and H3f3a **(D)**. Error bars indicate standard deviation (n=3). Astrisk indicates a significant difference (P<0.05).

Yeast two-hybrid screening was performed to identify proteins that are capable of interacting with the novel protein. Fbxoo protein fused with the GAL4-DNA binding domain was used as a bait protein to screen an activation domain-tagged cDNA library from rainbow trout oocytes. Multiple yeast clones were selected as a result of their growth on high stringency plates (SD/-ade/-his/-leu/-trp). Three positive clones were finally identified as they showed *LacZ* activity in a β-galactosidase colony-lift filter assay (Figure 5A). The plasmids from these yeast clones were rescued and the interactions were verified by co-transformation of the rescued plasmids with the Fbxoo bait plasmid into yeast competent cells. DNA sequencing analysis of the rescued plasmids revealed that the plasmids contain sequences encoding nearly complete proteins for tissue inhibitor of metalloproteinase 2 (Timp2), adenylate kinase 2 (Ak2) and histone H3.3 (H3f3a). To test the binding affinity of the identified proteins with Fbxoo protein, a β-galactosidase CPRG assay was performed. As shown in Figure 5, the β-galactosidase activities in yeast cells harboring Timp2 (Figure 5B), Ak2 (Figure 5C) and H3f3a (Figure 5D) were all significantly higher than the control cells.

## Discussion

In the present study, we report the cloning and expression analysis of an oocyte-specific gene encoding a novel F-box protein in rainbow trout. We show that the expression of both *fbxoo* mRNA and protein is high in oocytes at early pre-vitellogenesis stage, suggesting an important role for this protein in early oocyte development. The novel protein does not contain any of the common secondary motifs and therefore it belongs to the family of FBXO proteins. It is the first oocyte-specific F-box protein identified in a fish species.

In mouse, an oocyte-specific F-box protein gene, *Fbxw15/Fbxo12J*, has been reported [16]. The gene was identified by microarray analysis as the top increaser of 24 genes showing increased expression in neonatal mouse ovaries at 48 h and 96 h relative to < 24 h after birth. The oocyte-specific F-box protein is believed to play a role in the regulation of oocyte development based on its specific expression in oocytes and its expression pattern during follicular development [16]. Despite the fact that FBXW15/FBXO12J is also an F-box only protein [17] and is exclusively expressed in oocytes, it is not an orthlogue of Fbxoo as the two proteins share only 20% protein sequence identity. The fact that Fbxoo only shows high sequence similarity to an uncharacterized protein in zebrafish and Xenopus indicates that Fbxoo is likely fish/amphibian specific. This speculation is supported by synteny analysis showing that the corresponding loci of the zebrafish gene on the syntenic regions of human chromosome 6 and mouse chromosome 10 do not code for any genes (data not shown).

In growing oocytes, some transcripts undergo deadenylation in the cytoplasm where they are packaged into messenger ribonucleoprotein (mRNP) particles [18,19]. During oocyte maturation, cytoplasmic polyadenylation extends the poly (A) tail of these transcripts, a process associated with their timely translation [20,21]. The presence of a typical U-rich cytoplasmic polyadenylation element (UUUUUAA) in the 3′UTR of *fbxoo* mRNA indicates that this gene may have controlled translation during oocyte maturation. In addition, Fbxoo is predicted to have a SUMO site located on lysine residue 190, indicating that the function of Fbxoo protein may be regulated by sumoylation, a post-translational protein modification process known to change and regulate the function of a protein [22]. Sumoylation of oocyte-specific proteins have been reported previously. For example, POU5F1, an oocyte-specific transcription factor, is sumoylated [23] and sumoylation of POU5F1 leads to increased stability, DNA binding, and transactivation function of the protein [24].

Many F-box proteins are located in both cytoplasm and nucleus [25]. Our results also show that the expression of *fbxoo* mRNA and protein is detected in both cytoplasm and nucleus of oocyte at early developmental stages. While many F-box proteins are ubiquitously expressed [25], tissue specific F-box proteins with specific functions have been reported [26-28]. In particular, muscle-specific F-box proteins such as ATROGIN-1 and MURF1 are known to have unique functions in the degradation of important regulatory proteins during muscle atrophy [27,29]. As an oocyte-specific F-box protein predominantly expressed in early pre-vitellogenic and early vitellogenic oocytes, it is reasonable to believe that Fbxoo plays a crucial role during the early oocyte growth in rainbow trout.

F-box proteins bind to their specific substrates for ubiquitin-mediated proteolysis. They often have additional carboxy-terminal motifs (e.g. WD repeats and leucine-rich repeats) capable of protein-protein interaction. As an FBXO protein, Fbxoo does not have the common secondary motifs but may contain potential protein-protein interaction domains not yet identified. As an attempt to understand the function of the novel protein, we screened for Fbxoo-interacting proteins using the yeast two-hybrid system and identified three proteins including Timp2 as potential substrates of Fxboo protein. TIMP2 is a member of the TIMP family proteins known to play an important role in regulating the activity of matrix metalloproteinases (MMPs), which are the key metal-dependent enzymes in the extracellular matrix remodeling of follicular tissue. TIMPs are highly abundant in reproductive tissues and are well known to be involved in follicular development and early embryogenesis in a number of mammalian and fish species [30-32]. The expression of *Timps* has been detected in unfertilized oocytes, zygotes, cleavage stage embryos and blastocysts as well as in granulosa and theca cells [33]. The functional interactions between TIMPs and MMPs have been thoroughly investigated during folliculogenesis, whereas much less is known about the post-secretory mechanisms regulating the activity of TIMPs [34]. As a TIMP2 binding partner involved in the ubiquitin proteosome system, Fbxoo may regulate the post-secretory activity of Timp2 to modulate oocyte development in rainbow trout.

## Conclusions

We have cloned a novel oocyte-specific gene encoding an F-box only protein in rainbow trout and demonstrated that it is abundantly expressed in early pre-vitellogenic oocytes. Our results suggest that Fbxoo may play an important role in early oocyte development by regulating other critical proteins involved in oogenesis in rainbow trout.

## Competing interests

The authors declare that they have no competing interests.

## Authors’ contributions

LW, SKT and WW performed the experiments. LW drafted the manuscript. CER and JY designed the study, supervised the experimental work and revised the manuscript. All authors read and approved the final manuscript.

## Supplementary Material

Additional file 1: Figure S1 Complete cDNA and deduced amino acid sequences of rainbow trout Fbxoo. The F-box domain is shaded in grey and the predicted Sumo site is shaded in blue. The sequence of the peptide used to produce anti-Fbxoo antibody is boxed. The start codon (ATG) and stop codon (TAG) are highlighted in yellow. The polyadenylation signal (AATAAA) and the cytoplasmic polyadenylation element (TTTTTAA) are both underlined and bolded. The cDNA sequence has been deposited in the NCBI database with the accession number: HQ201417. Click here for file
