# Supplementary material for: Cloning and characterization of a novel oocyte-specific gene encoding an F-Box protein in rainbow trout (Oncorhynchus mykiss)
Source: Reprod Biol Endocrinol. 2013 Sep 4;11:86. doi: 10.1186/1477-7827-11-86 (PMC3846697; doi:10.1186/1477-7827-11-86)
Supplement: Additional file 1: Figure S1 — Complete cDNA and deduced amino acid sequences of rainbow trout Fbxoo. The F-box domain is shaded in grey and the predicted Sumo site is shaded in blue. The sequence of the peptide used to produce anti-Fbxoo antibody is boxed. The start codon (ATG) and stop codon (TAG) are highlighted in yellow. The polyadenylation signal (AATAAA) and the cytoplasmic polyadenylation element (TTTTTAA) are both underlined and bolded. The cDNA sequence has been deposited in the NCBI database with the accession number: HQ201417. [file 1477-7827-11-86-S1.rtf]

Additinal fie 1, Supplemental figure S1


1    acaggtggcaaatATGGCACTTCGTGAGGATAGTTCGGAGGATGAGTTGCAGGGACAGTTCTCGCTTTTA 70   
                      M  A  L  R  E  D  S  S  E  D  E  L  Q  G  Q  F  S  L  L 
71   TCCGCGACTTCATTCGAGGTCACAGATGAAAGTGAGGACACACATGGACGCTCCGAAACGGGGATACTAG 140  
       S  A  T  S  F  E  V  T  D  E  S  E  D  T  H  G  R  S  E  T  G  I  L 
141  ACTTAAGTGACGAAGTCTTTATTCTCATCCTACGACGGCTGGATCCCACGTCTCTCTTGAGAGTTGGGAG 210  
      D  L  S  D  E  V  F  I  L  I  L  R  R  L  D  P  T  S  L  L  R  V  G  S 
211  CACCTGCCGAACCCTGTTTCGAGTTTGTTCCTGCAACTCACTGTGGACAAAACACTTCCAGACCTCATTT 280  
       T  C  R  T  L  F  R  V  C  S  C  N  S  L  W  T  K  H  F  Q  T  S  F 
281  GGAGTCCCGTTTGCCACCGCCGCCTGCTCCATCTCTGCCAAGAGTGCCTTCCGTTTGGTCTTCATGTGGC 350  
      G  V  P  F  A  T  A  A  C  S  I  S  A  K  S  A  F  R  L  V  F  M  W 
351  GAACTCTCTTTAGAAACCTGCATTGTAACCGGTCTCTTCAGGAGAAGCTCTTTGCAGAGATCCCATTCCC 420  
      R  T  L  F  R  N  L  H  C  N  R  S  L  Q  E  K  L  F  A  E  I  P  F  P 
421  ACCACACAAGTACTGGGTCCAGTGGCTGGTTCTGGAAGAGACTGTTCCTCTGCCCTCCGTGAGACTGCCT 490  
      P  H  K  Y  W  V  Q  W  L  V  L  E  E  T  V  P  L  P  S  V  R  L  P 
491  TGCACCGACATAGAGAGCTTATGGGGAATTGAGAAGGAAGTGTTGGAAGGTAAAGTCCAAGAGAAAGATG 560  
      C  T  D  I  E  S  L  W  G  I  E  K  E  V  L  E  G  K  V  Q  E  K  D 
561  AGGATGAGGGCAGAATGCTGAAGTTCGAATGGAAGGAGCTGTATGCCTTGGCCCTTGAGCACCATGGAAG 630  
      E  D  E  G  R  M  L  K  F  E  W  K  E  L  Y  A  L  A  L  E  H  H  G  S 
631  CATTGCCAAGGTTTTCCAGCATGTTCTCAACCAACAGAGCAATGACCACTGTGAGCTGGAGGCCATGTTC 700  
      I  A  K  V  F  Q  H  V  L  N  Q  Q  S  N  D  H  C  E  L  E  A  M  F 
701  AGTCAGTACAGCCAGTGTCGGTTCCAGTGGCTCTTCACCTACTGGTTGTTCCGCCAGCCAGCGCCCTTCG 770  
      S  Q  Y  S  Q  C  R  F  Q  W  L  F  T  Y  W  L  F  R  Q  P  A  P  F 
771  ACAGGCAGCTCAGGGCCATCTACCTGCAGTGGCAGAAGCACAGCAAGAGGAAGGTGGTGTCCTGGGGAGG 840  
      D  R  Q  L  R  A  I  Y  L  Q  W  Q  K  H  S  K  R  K  V  V  S  W  G  G 
841  CACGTTGTGTGACATCAGATACCTGGCCTCATTACATCACATCACTTCCGACTACTGGCGGGGCAAGCTG 910  
      T  L  C  D  I  R  Y  L  A  S  L  H  H  I  T  S  D  Y  W  R  G  K  L 
911  GCCCAGGGTGACGAGACTGTGGGAATTCAGACAGTGGAAAACTATTTCTCCATGTGCAAATCCCTGGTGG 980  
      A  Q  G  D  E  T  V  G  I  Q  T  V  E  N  Y  F  S  M  C  K  S  L  V 
981  CCTGGATTTTAGGGCGTGACTGGGGCAGATTGAAAAGCAAAAAGGTGTACGAGGACACGCTGGAGGGTGT 1050 
      A  W  I  L  G  R  D  W  G  R  L  K  S  K  K  V  Y  E  D  T  L  E  G  V 
1051 GTACCTGCTGCTGAGGAGGGAGATGCAGGAGACCCTGGTGGAGCACGAGAGGTTCTGGCAGGTGGCCAAG 1120 
      Y  L  L  L  R  R  E  M  Q  E  T  L  V  E  H  E  R  F  W  Q  V  A  K 
1121 GTCCAGATGACCCGAGTGTGTACCCTGGAGGAGACTGCTGTAAACTATGTCAACTGGAAGATGATTGAAA 1190 
      V  Q  M  T  R  V  C  T  L  E  E  T  A  V  N  Y  V  N  W  K  M  I  E 
1191 CACTGCCCTACTACAAGCTGTACTTGGTGTCAGGCAACATGGTTTACCTAGACCACGTGCAGGGCTTCCT 1260 
      T  L  P  Y  Y  K  L  Y  L  V  S  G  N  M  V  Y  L  D  H  V  Q  G  F  L 
1261 CCACAGGAAGAGGCTGGTCCATGACTGGTTCTTCATGAAGGAGAACACCTGGGTCAGACAGCTGCTGCCT 1330 
      H  R  K  R  L  V  H  D  W  F  F  M  K  E  N  T  W  V  R  Q  L  L  P 
1331 GGGGACCTCTACCCTCTGCTGGAGTTTGACACCAAGATCTCCCAAGACAGCCTGCATGGAGACTCCATGC 1400 
      G  D  L  Y  P  L  L  E  F  D  T  K  I  S  Q  D  S  L  H  G  D  S  M 
1401 CAGCCCAGCTGAGCAGGGTGATGTGGCTGTACCTCCACTCTGGACAGACACTCTACCTGGAGGCAGTGAA 1470 
       P  A  Q  L  S  R  V  M  W  L  Y  L  H  S  G  Q  T  L  Y  L  E  A  V  K 
1471 AGGCCTGGTGCTGCAGTGTGCTCAGGCCAGTCTGGGACACTTCTGCAGCCTCTCCCCTGGTGCCTCTATT 1540 
      G  L  V  L  Q  C  A  Q  A  S  L  G  H  F  C  S  L  S  P  G  A  S  I 
1541 CAGCCCCATGTCAGCTAGctggctaacgcagattgagcactctgttttagagcctctacgagatgtacag 1610 
      Q  P  H  V  S  *  
1611 tcccatgttgttgttctttactggatccagtgtccagtgcttttatcactttttactgtattttatcacc 1680 
1681 actcagattttagctggcgagctggaattaatagattttaaatgaatttttaaacttgaatctttatcaa 1750 
1751 tagttaatgaaatgtaggctattgatgtacattgtagaaaccgtgtttgctgtcatgtaattactctaca 1820 
1821 gccaacatttggggcaagctattgaagtgatgacattgctcaatcattctcaaatggtaaatgtgtcata 1890 
1891 ttcaaagcctaacaattcttggccaattaacaggctagaaacttaacagtgctgggttgctgtataatga 1960 
1961 ataaacaactctgacaaacttaaaaaaaaaaaaaaa 1996 
